# Arterial switch operation in patients with Taussig - Bing anomaly - our experience in coronary anatomy and staged repair

**DOI:** 10.1186/1749-8090-10-S1-A187

**Published:** 2015-12-16

**Authors:** Sadavisan Ilango, Nakao Mazakasu, Loh Yee Jim, Ong Kim Kiat, Jonathan Choo, Marielle Fortier

**Affiliations:** 1 Cardiothoracic Surgery, K.K.Women and Children's Hospital, 229899, Singapore; 2 Cardiology, K.K.Women and Children's Hospital, 229899, Singapore; 3 Radiology, K.K.Women and Children's Hospital, 229899, Singapore

## Background/Introduction

A variety of definitive operations have been used to manage patients with Taussig Bing anomaly. But arterial switch operation and closure of the VSD is the most widely used procedure.

## Aims/Objectives

This study analyzes the impact of the position of the coronary arteries and the feasibility of staged surgical approach in children with Taussig Bing anomaly with aortic arch obstruction.

## Method

From 2013 to 2015, 4 patients were presented with Taussig Bing anomaly. Among four, one patient had Taussig Bing anomaly and coarctation of aorta with 1 left circumflex and 2 right sided coronary artery, another had intramural origin of right coronary artery and left coronary artery, both of which were arising from left facing sinus. The third patient had an usual coronary pattern, and fourth infant had single coronary artery arising from the left anterior aspect of the aortic root.

## Results

Seventy five percentage of the patients underwent arterial switch operation with right and left coronary artery transfer to neoaorta. Coarctation repair was performed prior to arterial switch for the patient who had Taussig Bing with coarctation of aorta. Third patient was proceeded with Blalock Taussig shunt followed by bidirectional Glenn operation as the coronary artery was running below the pulmonary annulus. PA band was applied first and ASO was performed subsequently for the patient who had single coronary anatomy. There was no death. One patient required reoperation for aortic arch obstruction. Others had no significant complications.

## Discussion/Conclusion

Arterial switch operation and ventricular septal defect closure in neonates and young infants have yielded an excellent outcome for Taussig Bing anomaly. In the presence of aortic arch obstruction, staged arch reconstruction followed by early intracardiac repair had given good result. We would consider two-stage repair in cases of complex anatomy. Single coronary artery with single ostium can be transferred with trap door technique with excellent results.

